# A Revised Classification and Treatment Algorithm for Acquired Split Earlobe, With a Description of the Composite Technique and its Outcome

**DOI:** 10.7759/cureus.10422

**Published:** 2020-09-13

**Authors:** Kalesh Sadasivan, Ajayakumar Kochunarayanan

**Affiliations:** 1 Plastic and Reconstructive Surgery, Government Medical College, Thiruvananthapuram, IND

**Keywords:** aesthetic surgery, earlobe repair, algorithm

## Abstract

A new classification for acquired split earlobes based on surgical implications is proposed here. Numerous techniques have been described and the choice of the surgery is a difficult one so a simplified approach to the choice of the repair method is provided. Any surgical repair, to stand the test of time must be simpler to perform and should serve the purpose of aesthetics as well as function. The composite method of earlobe repair described here incorporates an upper triangular flap preserving the perforation; the undermining of cleft margins to prevent suture line grooving, and a lower L-plasty to prevent inferior notching. This technique is easy to understand and perform, and avoids a second appointment and visit for ear piercing.

## Introduction

Repair of torn earlobe is a frequently performed aesthetic surgical procedure [1]. Torn earlobes are clinically referred to as split earlobes or cleft earlobes [2]. The etiology of the condition may be trauma, heavy earrings or even metal allergy [3]. The incidence of the condition is about 1-2% [4]. Split ear-lobes or earlobe clefts are classified as congenital and acquired [5], and the latter are classified as complete and incomplete [5,6]. Many classifications and techniques have been described for repair of various grades of split earlobes. Agarwal and Chandra classification [7] and Blanco-Davila and Vasconez classification [8] are the commonly used ones. When the commonly used classifications of split earlobe are analysed, the first two types of both the classification are incomplete clefts and the third is a complete cleft. Agarwal and Chandra have incorporated two more types of complete cleft with fibrosis of the tissue or multiple splits and loss of tissue [7].

The Blanco-Davila and Vasconez classification has two major classes, namely the incomplete split lobe and the complete split lobe [8]. The incomplete split lobe is further classified as Type I, where the boundaries of the cleft extend less than half the distance between the original piercing and the inferior margin of the earlobe, and Type II in which the boundaries of the cleft extend more than half the distance between the original piercing and the inferior margin of the earlobe. The complete split lobe in this classification is also called as the Type III, where the cleft perforation completely splits the earlobe.

In Agarwal and Chandra classification, the classification is as incomplete split lobe and complete split lobe [7]. Here, the Type I refers to the stretched-out perforation of earlobe more than twice the original diameter and the Type II comprises near total split of earlobes, thin rim of soft tissue remaining; both are incomplete splits. The complete split lobe is divided into Type III with the cleft perforation completely splits the earlobe, Type IV having total split of earlobe with preservation of soft tissue, and the Type V with total split of earlobe with fibrosis and retraction of earlobe halves.

We can see that the aesthetics of the inferior margin is not addressed and the surgical implications of the classifications are not well clear. Thus, we suggest a modification of the existing classification, with incorporation of two subclasses in Type I and II of incomplete split earlobe deformity and two subclasses of complete split with surgical implications and the recurrent split earlobe.

Many techniques have been described for repair of various grades of split earlobes - the straight-line repair with excision of the original perforation as in McLaren [9], Apesos and Kane [10] or with preservation of the original perforation as in Boo-chai [5] and Buchan [11]. The problem in straight line repair is the grooving along the closure line and the inferior notching or bulge. Pardue repair and its modifications - the basic idea behind Pardue and similar designs - are to preserve the original perforation in full or part or form a new epithelium lined one. A flap from the upper posterior cleft margin that rotates to form the new perforation can achieve this. The lower end is closed by straight line as in Pardue [6], Patrocínio et al. [12] and Ribeiro et al. [13], or a Z-plasty is incorporated, as in Hamilton and La Rossa [14], Walike and Larrabee [15], and Fayman [16]. Elsahy used similar flaps from both sides of the upper cleft and simple closure was done [17]. V-plasty-based repairs - Kalimuthu et al. [18] - incorporated V-plasty where a 3-mm “V”-shaped flap is made on one flap that fits into a slit of same size on opposite flap. L-plasty and similar repairs - in Fatah [19] - L-shaped flap on anterior half of the lobe that fits into a same defect made on the posterior half of lobe (anterio-posterior direction). This may be combined with Pardue flap. In Harahap [20], an L-shaped flap was designed on one half of the lobe that fits into the same defect made on the other half of lobe (medio-lateral direction). Fearon and Cuadros also used this L-plasty method [21]. Z-plasty techniques - Tromovitch et al. [22] - used full thickness Z-plasty while Reiter and Alford [23] advocated Z-plasty anterior part of the closure and posterior part was directly closed. Antero-posterior opposing flaps - these are much technically demanding procedures and were used by Effendi [24], Arora [25] and Reiter and Alford [23]. The triangular flap, in this technique, was used by Argamasso [26] and it preserved the primary perforation and the rectangular flap technique - popularized by Zoltie [27] where the original perforation was preserved.

Regarding the types of cleft, the partial clefts can be repaired using the techniques used by McLaren [9], Tan [28], Reiter and Alford [23], Abenavoli [29] and Vujevich et al. [30]. The complete clefts can be corrected by techniques described by Apesos and Kane [10], Hamilton and La Rossa [14], Reiter and Alford [23], Fatah [19], Harahap [20], Kalimuthu et al. [18], without preservation of the primary perforation, or the perforation may be preserved using Boo-chai [5], Pardue [6] and its modifications, Zoltie [27], Argamasso [26], Elsahy [17], and Ribeiro et al. [13] techniques. Some described techniques do not preserve the primary perforation, converts an incomplete cleft to complete thus violating a normal bridge of tissue or are technically demanding. The choice of procedures depends upon the need to preserve the original perforation due to aesthetic reasons, to avoid another ear-piercing procedure, the type of earlobe cleft and the need to eliminate bulging or notching at the inferior border. The ideal repair is one, which has a perforation incorporated at the upper end, middle scar without groove and lower edge without notching. This can be accomplished by incorporating a Pardue flap at the upper end, undermining the middle part before suturing and Z-plasty or L-plasty at the lower end, with or without converting the incomplete cleft into a complete one. It is understandable that the natural contour of the inferior margins, if lost, by trauma or surgery, it cannot be regained with any surgical procedures. Therefore, we need to maintain a restraint in violating a natural inferior border. In addition, the scar, on the inferior end can cause retraction and notching or bulging on the site, resulting in aesthetic compromise. There have been incorporations of Z-plasty, L-plasty or other modifications like lap-joint technique to address this contour irregularity at the lower end [19], but results have been variable. The patient has to depend on hiding the scar under earrings. The myriad of techniques and the complexity of the procedures make choice of an ideal technique difficult. Considering the above by incorporating the best components from the historical techniques, we suggest this new technique of split earlobe repair. The objective of the study is to describe the composite method of split earlobe repair, observe its outcomes and to formulate a revised classification with an algorithm for surgical repair of split earlobes.

## Materials and methods

We studied 75 randomly selected post traumatic split earlobes (both complete and partial split earlobes), that were operated in the plastic surgery unit since the year 2011, using the composite technique (see below). We had assessed our patients at 12 months after the procedure, according to our unit protocol. The lower contour abnormalities like inferior notching and inferior bulging, the vertical suture line grooving, and recurrence were analyzed. The permission from the Institutional Research Committee and the Human Ethical Committee to publish our patient data since 2011 was obtained (IEC No.08/08/2017/MCT).

## Results

Revised classification

**Figure 1:**
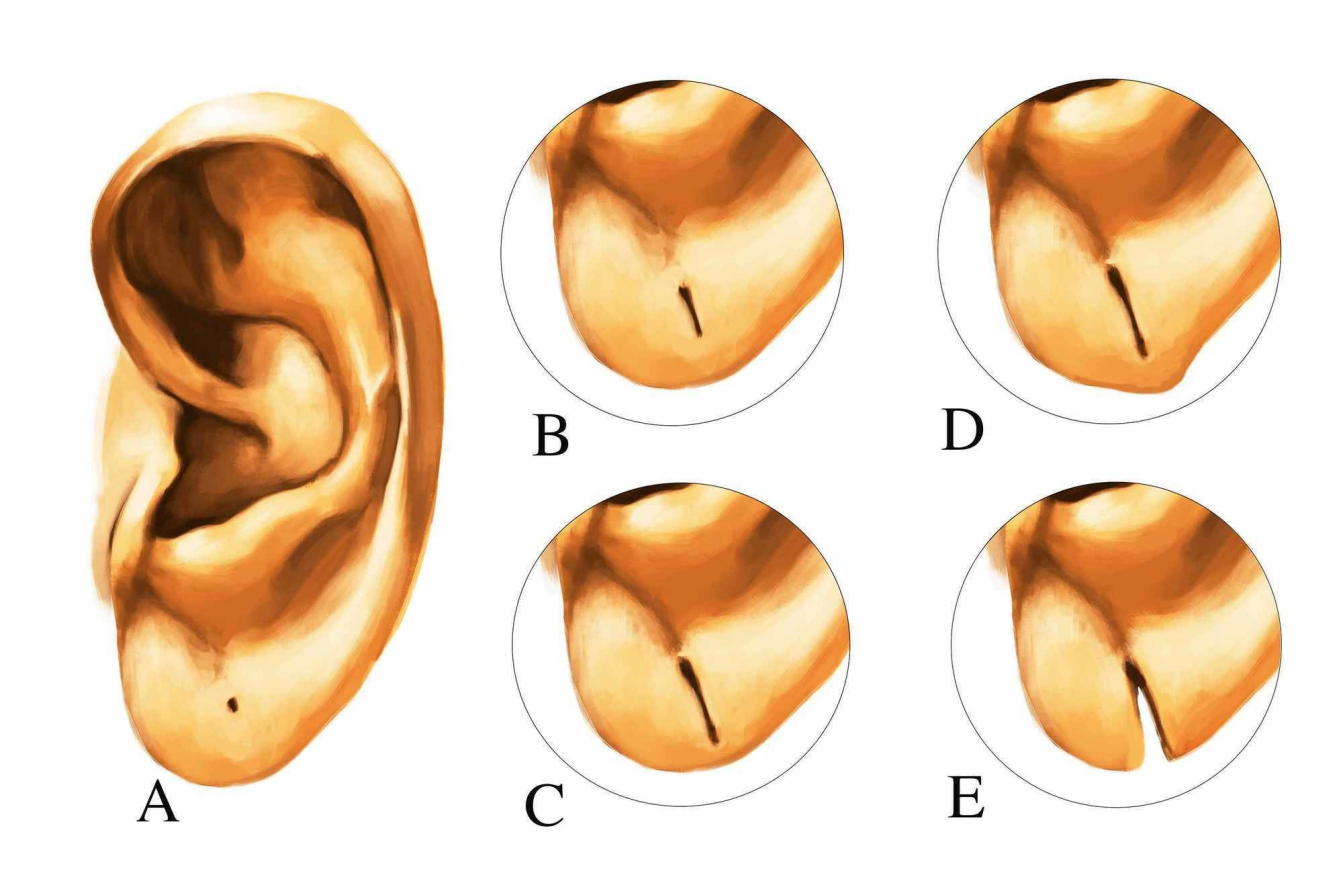
Revised classification of split earlobes A: Normal earlobe B: Type I: Elongated perforation with length less than that of normal tissue measured from lower end of the cleft to the inferior margin of the earlobe C: Type IIA: Elongated perforation with length more than that of normal tissue measured from lower end of the cleft to the inferior margin of the earlobe D: Type IIB: Inferior contour abnormal, ptosis of earlobe present E: Type III: Total split of earlobe

The revised classification of incomplete split earlobes has three major types. This new classification also incorporates two subclasses within each of them. The sub-classification in Type I & II is based on the presence of ptosis and whether the normal aesthetics and convexity of the inferior margin is preserved (Figure 1); and that in Type III it is based on whether primary repair is possible or some local flaps are needed. Thus, the new classification has surgical implications. This system can be applied for recurrent cases too (Table 1).

**Table 1:** Revised classification of split earlobes

Type of Split Earlobes	Sub-types
Type I: Elongated perforation with length less than that of normal tissue measured from lower end of the cleft to the inferior margin of the earlobe	IA: Inferior contour normal, no ptosis of earlobe
	1B: Inferior contour abnormal, ptosis of earlobe present
Type II: Elongated perforation with length more than that of normal tissue measured from lower end of the cleft to the inferior margin of the earlobe	IIA: Inferior contour normal, no ptosis of earlobe
	IIB: Inferior contour abnormal, ptosis of earlobe present
Type III: Total split of earlobe	IIIA: Total split of earlobe with preservation of soft tissue with or without ptosis
	IIIB: Total split of earlobe with loss of soft tissue or retraction of split ends needing local flaps for repair
Recurrent Cleft earlobe: Any of the above suffixed with ‘r’	

The composite technique of repair

**Figure 2:**
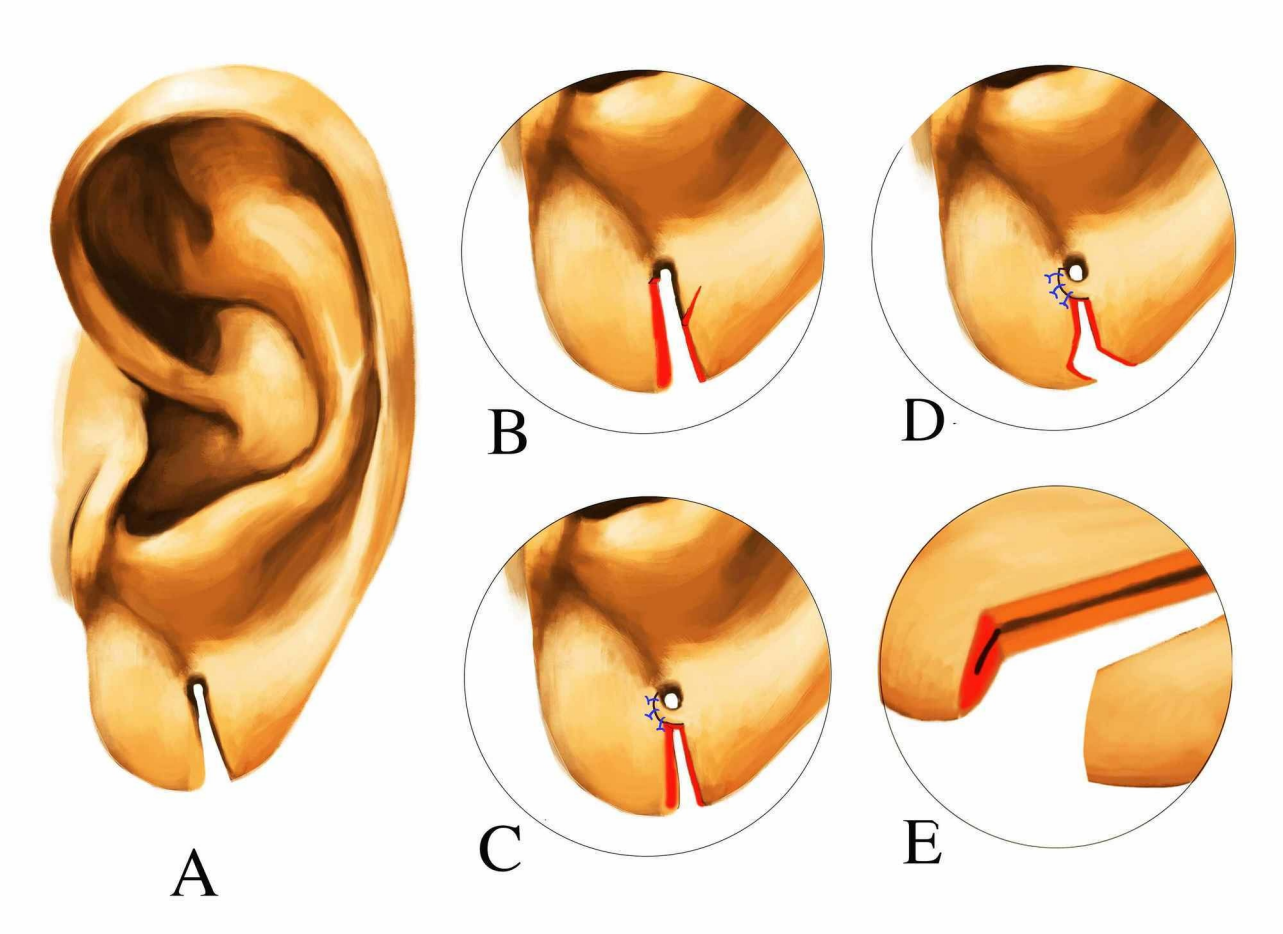
The composite technique of repair A: Split ear lobule B: Freshening of edges and making the triangular flap C: The triangular flap is sutured onto the freshened anterior edge of the cleft D: L-plasty at lower end for contour irregularity or ptosis E: Undermined straight-line closure in two layers

The procedure is done under Great Auricular Nerve block supplemented with local infiltration, with Loupe magnification of 4x. The anterior edge of the cleft and the lower quarter of the original perforation are excised. We preserve a similar tissue on the posterior margin of the cleft and the posterior rim of the original perforation. A full thickness triangular flap, with its base near the original perforation, is fashioned out from the upper part of the posterior margin of the cleft. The length of this flap is determined by the size of the new perforation needed (Figure 2B). This Triangular flap (T) is checked for the vascularity by freshening the tip. The flap is sutured onto the freshened anterior edge of the cleft and the lower quarter of the original perforation, over a stent (No. 16 Grey cannula sheath) to form the perforation (Figure 2). To prevent this stent from slipping the free ends sutured to each other. Thus, a new perforation is created lined by normal skin from the proximal and lateral cleft margin. An undermining is done for about 3 mm in the mid-substance of the lobe to facilitate an edge eversion during closure with 6-0 Polypropylene mattress sutures (Undermined Straight-Line Closure) (Figure 2E). These skin sutures were removed on fifth postoperative day. The cannula sheath is retained, with its ends cut, to continue the stent effect for three weeks. The patient is advised to wear a weightless earring after removing the stent from three to six weeks. And normal earrings are advised to be worn after six weeks. In this technique, the incomplete cleft is treated as such, and is not converted into a complete cleft as in some conventional methods. The convex and natural inferior contour of the earlobe is not interfered with. If there is any significant, contour irregularity or ptosis pre- or post-operatively we incorporate an L-plasty at lower end to correct it, as it is much simpler than a Z-plasty (Figure 2D, 2E).

Seventy-five repaired earlobes were followed up over a time of 12 months for recurrence, and aesthetic abnormalities of inferior notching, inferior bulging and suture line grooving. The scar quality was not assessed. Twenty-four patients had bilateral and 27 patients unilateral cleft earlobe repairs, all were female patients. Inferior notching was observed in two cases (2.6%) and was detected by the authors than the patient. The incidence of this complication was almost equal in Type II (3.3%) and Type III (3.0%). Inferior bulging was recorded in three cases (4%). The patient was oblivious of this and was usually hidden by the earring. Suture line grooving was seen in two cases (2.7%). Recurrence was recorded in three cases (4%) during the follow-up period. Of these three patients with recurrence, two of them were using ornaments made of metals other than gold and the third patient had a recurrence because of trauma (Table 2).

**Table 2:** Types of split earlobes and complications observed in the study

Type of cleft	No of cases	Complications over 12 months post-operation (IIIB requires local flaps and hence is excluded from this analysis)			
		Inferior notching	Inferior bulging	Suture line grooving	Recurrence
I	7	0	0	0	0
II	30	1	1	1	1
III	33	1	2	1	2
Total	75	2	3	2	3

## Discussion

Pardue mentioned that if the split is incomplete, the bridging tissue should be excised, creating a complete cleft, to avoid bulging of the bridging tissue [6]. It is also observed that in many classical techniques for correction of incomplete clefts, the perforation is converted into a complete one to surgically repair it. This is unnecessary especially in elongated perforations and Type IIA of earlobe cleft in our classification. Thus, in a definite subset of incomplete cleft the repair can be accomplished without conversion into complete cleft, and that the bulging can be avoided in them by conserving the amount of tissue excised on the cleft margins. Thus, it is to be carefully decided on a technique that does not compromise on the natural lower contour of the ear lobule.

Generally, in Type I cleft, the Type IA is common, the perforation is elongated, but the inferior contour is normal. In these cases, we need to preserve the natural lower border and the perforation. Only very few patients will want surgery at this stage and mostly is demanded by the patient, with a higher degree of cleft on the opposite ear surgery, as a part of bilateral procedure. In Type IA we prefer to do upper triangle flap with conservative excision of the cleft margins and undermined closure. In all Type IB or in case of Type IA with post-closure inferior margin bulge (very rare), we perform L-plasty at the lower end.

In Type IIA we treat exactly as in Type IA with upper triangle flap and undermined cleft closure. In Type IIA with post-operative bulge and in Type IIB, where there is ptosis, we incorporate L-plasty at the inferior end. Minor grades of bulge at the inferior end gradually settle with time. In Type IIIA we treat with upper triangle flap, straight-line undermined closure and L-plasty at the end to prevent notching and to correct ptosis if present. And in Type IIIB where there is a loss of tissue, we use local flaps (Table 3).

**Table 3:** Split earlobes and the algorithm for surgical repair

Types	Sub-types	Surgical repair methods
Type I cleft	IA	Upper Triangle flap + Undermined cleft closure
	IB	Upper Triangle flap + Undermined cleft closure + L-plasty at lower end
Type II cleft	IIA	Upper Triangle flap + Undermined cleft closure +/- L-plasty at lower end
	IIB	Upper Triangle flap + Undermined cleft closure + L-plasty at lower end
Type III cleft	IIIA	Upper Triangle flap + Undermined cleft closure + L-plasty at lower end
	IIIB	Local flaps: Bilobed /pre/post auricular flaps/other flaps
Recurrent cleft earlobe	Any of the above suffixed with ‘r’	Cartilage grafts or Non-absorbable buried suture in the sub-cutaneous plane.

Thus, it can be seen that this technique of repair can be used in all types of ear lobe clefts, except Type IIIB, which needs local flaps. This technique is an amalgam of various components used in already described methods of repair. The average operating time for this technique is 20 minutes. This technique incorporates the best components in the best combination. It is easy to understand and perform.

The composite technique uses all the best components for the optimum result. This new technique can be considered as a combination of upper triangle flap (modified from Pardue technique), undermined cleft repair [10] and L-pasty at lower end. The original Pardue flap is a long rectangular flap raised from the upper part of the posterior part of the cleft. This flap is very thin and often may have precarious vascularity in case of scarring, so we modified it into a wide based triangular flap. A new epithelium lined perforation is created with closure of the cleft and preservation of the aesthetics of the lower contour. Incorporating a flap strengthens the repair and is definitely a better support for the perforation than the scar of a straight-line closure. Excision of tissue must be minimal during the freshening of the cleft margin otherwise lower contour irregularity will follow. In case the repair results in inferior bulging an incorporation of L-plasty at lower part is mandatory. For recurrent cases, deep buried non-absorbable subcutaneous suture or cartilage grafts may be done, as suggested by other workers [7].

Straight-line technique for earlobe repair is the simplest and easiest. The cleft margins and the original perforation is excised and closed primarily without undermining or incorporation of Z-plasty. The patient is reviewed later for ear piercing, which is an obvious disadvantage. The new perforation is usually created away from the original one causing cosmetic unacceptability. The composite technique addresses all these issues. The complication rates for this method have also been presented here and comparative studies may be done to evaluate the superiority of the procedure.

## Conclusions

Any surgical repair, to stand the test of time must be simpler to perform and should serve the purposes of function and aesthetics. In split earlobe, the composite technique is easy to master and the three components achieve the function, the upper triangular flap preserves the perforation, the undermining prevents grooving and the L-plasty prevents inferior notching. The potential advantages of the composite technique are- preservation of the lower contour aesthetics of the split earlobe, an epithelium lined perforation recreated at aesthetically acceptable original site, avoidance of a second visit for ear piercing, the cuff of normal tissue at the lower pole supports the weight better than a full-length scar that can cause recurrence, the technique is easily mastered and reproduced and is extremely useful in a subset of incomplete cleft lobe without need for conversion into complete cleft.
